# Automated prediction of phosphorus concentration in soils using reflectance spectroscopy and machine learning algorithms

**DOI:** 10.1016/j.mex.2024.102996

**Published:** 2024-10-15

**Authors:** Fabio Eliveny Rivadeneira-Bolaños, Sandra Esperanza Nope-Rodríguez, Martha Isabel Páez-Melo, Carlos Rafael Pinedo-Jaramillo

**Affiliations:** a Escuela de Ingeniería Eléctrica y Electrónica (EIEE) Facultad de Ingeniería, Universidad del Valle, Cali, Colombia; b Departamento de Química (FCNE), Universidad del Valle, Cali, Colombia

**Keywords:** Prediction of total phosphorus concentration in soils using UV–VIS spectroscopy and machine learning, Neural networks, Macronutrient quantification, Total phosphorus, Precision farming, Machine learning, Reflectance spectroscopy, Soil analysis

## Abstract

A method is presented for predicting total phosphorus concentration in soils from Santander de Quilichao, Colombia, using a UV-VIS V-750 Spectrophotometer and machine learning techniques. A total of 152 soil samples, prepared with varying proportions of P_2_O_5_ fertilizer and soil, were analyzed, obtaining reflectance spectra in the 200 to 900 nm range with 3501 wavelengths. Additionally, 152 laboratory results of total phosphorus concentration were used to train the prediction model. The spectra were filtered using a Savitzky-Golay filter. Key wavelengths were identified using Variable Importance in Projection - Partial Least Squares (VIP-PLS) and Random Forest (RF), reducing the spectral bands to 1085. Principal Component Analysis (PCA) further reduced data dimensionality. A feedforward artificial neural network was then trained to predict phosphorus concentration. This method is faster than traditional lab tests by leveraging advanced data analysis and machine learning, offering results in less time. While sample preparation remains consistent with standard spectroscopic analysis, the value added by the proposed method lies in its data processing and interpretation. Currently applied to a single soil type, future improvements will include more soil types and other macronutrients, enhancing nutrient management in agriculture. Accurate macronutrient measurements aid in better fertilizer uses planning.

• Filtering spectra and determining relevant wavelengths using VIP-PLS and RF.

• Dimensionality reduction with PCA.

• Training feedforward artificial neural networks.

## Graphical abstract

**Figure:**
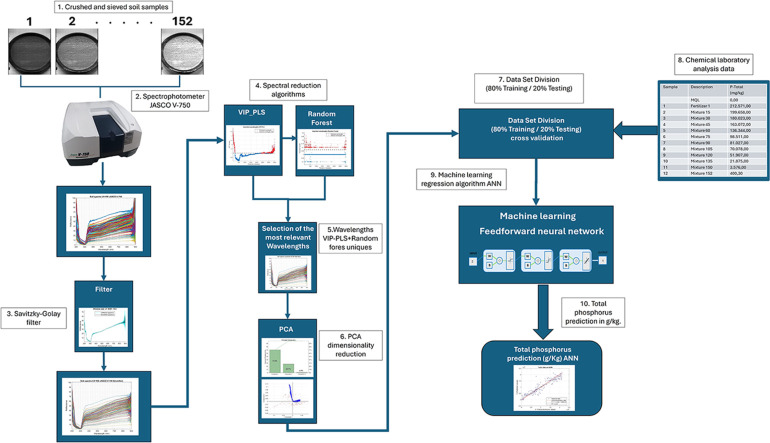

Specifications table

**Table:** 

Subject area:	Agricultural and Biological Sciences
More specific subject area:	Computer Vision and Pattern Recognition, Machine learning, Soil Science, Analytical Chemistry: Spectroscopy
Name of your method:	Prediction of total phosphorus concentration in soils using UV–VIS spectroscopy and machine learning.
Name and reference of original method:	
Resource availability:	https://data.mendeley.com/datasets/fvgswvt5ws/3 https://data.mendeley.com/datasets/n49xkddkgs/1

## Background

The motivation behind presenting this methodology lies in the need to advance the precision and efficiency of predicting the total phosphorus concentration in agricultural soils, a crucial factor for effective nutrient management in agriculture. In the region of Santander de Quilichao, Colombia, and many other agricultural areas worldwide, the availability of phosphorus in the soil plays a fundamental role in crop yield and environmental sustainability [1]. The use of the UV–VIS V-750^1^ Spectrophotometer and advanced machine learning techniques offers several significant advantages. First, it allows for the rapid and non-destructive acquisition of reflectance spectra from soil samples, covering a broad spectral range that provides detailed information about the soil's physical and chemical properties. This is crucial for assessing soil fertility and determining specific fertilization needs.

In recent years, reflectance spectroscopy and multivariate analysis have proven to be effective tools for the prediction of soil properties such as phosphorus content. Techniques such as PLS have been widely used due to their ability to handle large amounts of spectral data and their computational simplicity [2]. However, recent studies have shown that the integration of more advanced methods, such as Random Forest (RF) and neural networks, can overcome the limitations of linear approaches by capturing nonlinear relationships in spectral data [3]. Furthermore, the combination of VIP-PLS for feature selection and PCA for dimensionality reduction has been shown to improve the accuracy and computational efficiency of prediction models [4], enabling faster and less resource-intensive processing, which is crucial in large-scale studies.

Moreover, implementing techniques such as VIP-PLS, RF, and PCA optimizes the data analysis process by reducing the dimensionality of the spectra, identifying relevant features, and improving the accuracy of predictive models. This not only facilitates the interpretation of complex data but also accelerates the development of robust phosphorus prediction models.

The proposed methodology seeks not only to optimize the estimation of phosphorus concentration, but also to broaden its applicability and facilitate its use in different agricultural contexts. By upgrading standard spectroscopy equipment with advanced data analysis methods, access to technologies that were previously reserved for specialized and expensive software is broadened. This is particularly relevant for farmers and agricultural technicians in regions where resources are limited but the need for precise nutrient management is critical.

Finally, the inclusion of 152 laboratory results of phosphorus concentration in the model training underscores the robustness and validity of the proposed approach. However, the necessity to continue improving the model by incorporating more soil types and considering other macronutrients is recognized, ensuring even more effective and tailored agricultural nutrient management across different environmental and agricultural contexts.

## Method details

### Obtaining experimental data

**Fig. 1:**
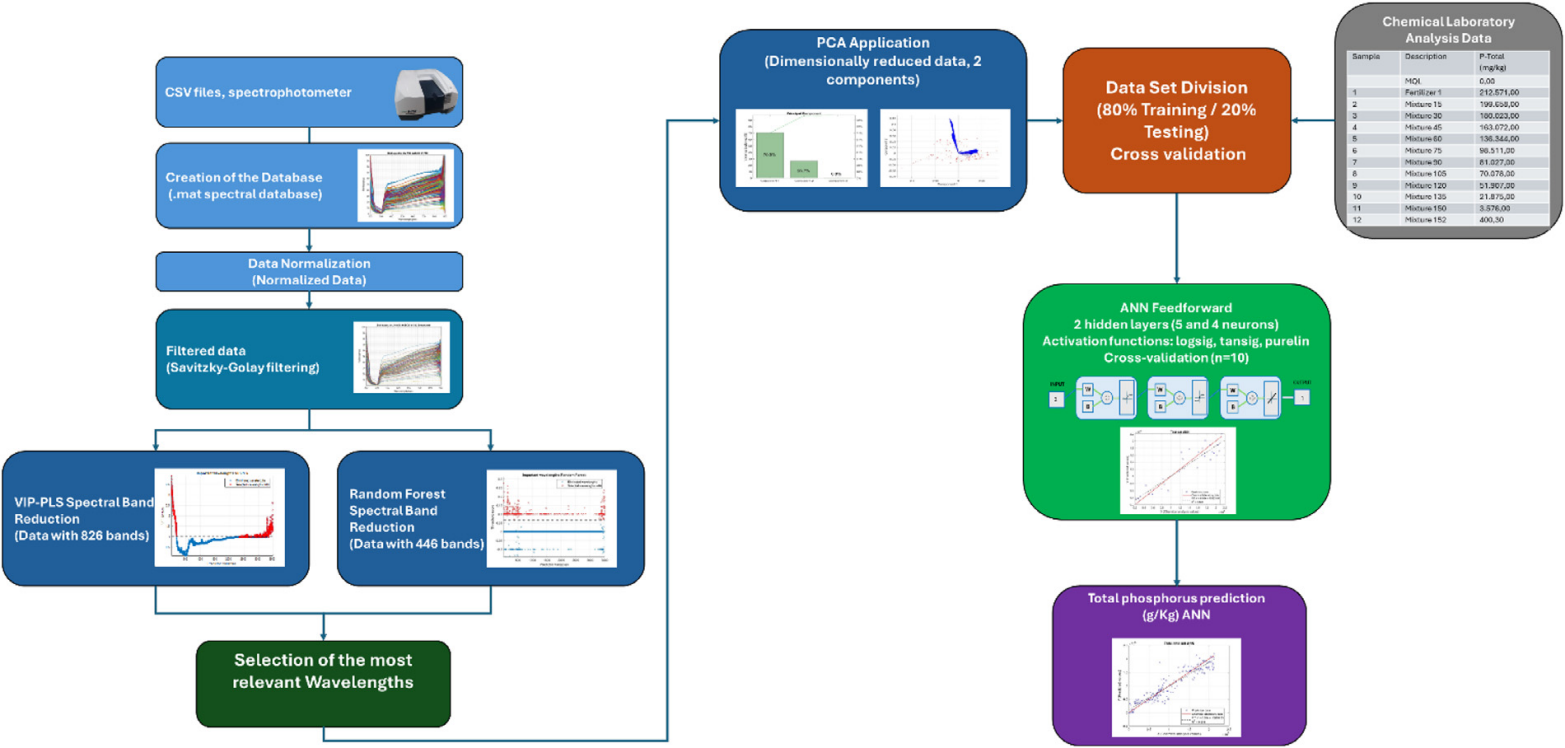
Block diagram of the method.

The structure of the designed method is illustrated in Fig. 1.

### Sample preparation and laboratory analysis

The soil sample preparation was carried out according to the procedure described by F. Rivadeneira [5], using air-dried acidic clay soil, characteristic of the region of Santander de Quilichao, Colombia. A soil-fertilizer ratio was used in which the diammonium phosphate (P_2_O_5_) fertilizer was proportionally reduced in each sample. Sample 1 consisted of 100% fertilizer (P_2_O_5_), while sample 152 contained only soil. The physicochemical properties of the soil are detailed in Table 1.

**Table 1:** Chemical analysis soil Santander de Quilichao. Reprinted from [5].

Soil Property Santander de Quilichao	
Clay (%)	64.8
Sand (%)	22.6
Silt (%)	12.6
Texture	Argillaceous
pH	4.3
CIC (cmol/Kg)	14.2
Interchangeable Aluminium (cmol/Kg)	4.58
MO (%)	4.17
CO (%) *	2.42
Humidity (%)	4.74

⁎ Total organic Carbon, CO (%) = MO (%) /1.74.

The total phosphorus concentration in the soil samples was measured through laboratory analysis using standardized methods for the chemical determination of soils, and Table 2 shows the phosphorus concentration for 12 samples.

**Table 2:** Proportions in the preparation of the soil samples. Reprinted from [5].

Sample	Description	P-Total (mg/kg)
	MQL	0.00
1	Fertilizer 1	212,571.00
2	Mixture 15	199,658.00
3	Mixture 30	180,023.00
4	Mixture 45	163,072.00
5	Mixture 60	136,344.00
6	Mixture 75	98,511.00
7	Mixture 90	81,027.00
8	Mixture 105	70,078.00
9	Mixture 120	51,907.00
10	Mixture 135	21,875.00
11	Mixture 150	3576.00
12	Mixture 152	400.30

This study [5] presents the results of these measurements in 152 samples collected from a geographically defined area in Santander de Quilichao (3°06′01.1″N, 76°30′59.9″W). Although this approach allowed for a controlled analysis of the soil-fertilizer interaction, we recognize that the lack of variability in soil types may limit the model's ability to generalize to other edaphic conditions.

From an academic perspective, this study provides an important contribution to the analysis of soils under controlled conditions. However, for broader applications, it will be necessary to include greater variability in soil types in future research in order to improve the robustness of the predictive model for different soil types. The data are available for download in the Mendeley Data repository.^2^

### Obtaining reflectance spectra

The reflectance spectra were obtained using a high-resolution UV–VIS JASCO V-750 Spectrophotometer. The most notable features of the equipment are detailed in Table 3.

**Table 3:** Specifications Spectrophotometer JASCO V-750.

V-750 UV–Visible Spectrophotometer	
Optical System	Czerny-Turner mount Single monochromator Fully symmetrical double beam type
Light Source	Halogen lamp, Deuterium lamp
Wavelength range	200 to 900 nm
Wavelength accuracy	±0.2 nm (at 656.1 nm)
Scanning speed	10∼4000 nm/min (8000 nm/min in preview mode)
Dimensions and weight	460(W) x 602(D) x 268(H) mm, 27 kg

**Fig. 2:**
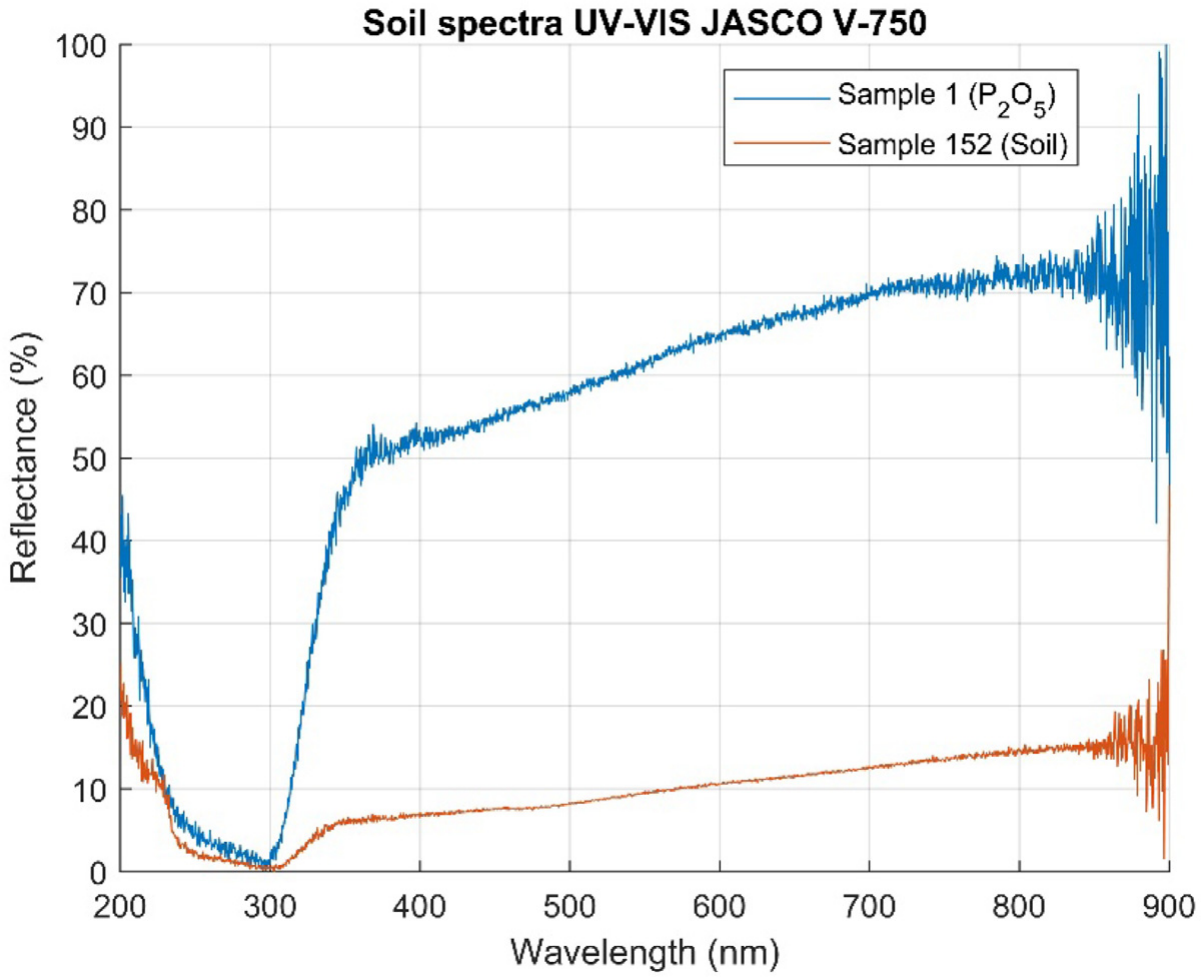
Soil Reflectance and P_2_O_5_ Spectra (200–900 nm).

**Fig. 3:**
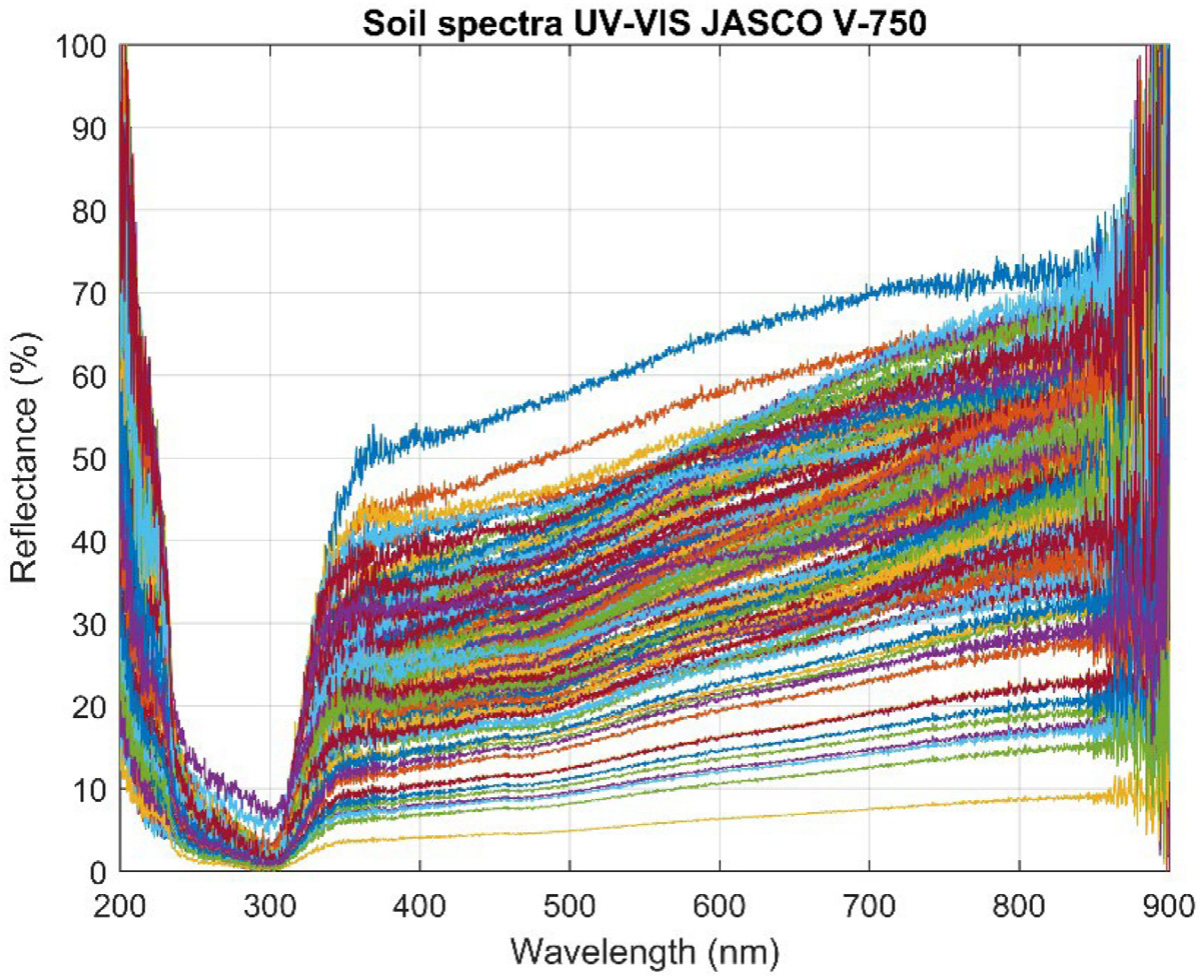
Database of reflectance spectra of 152 soil samples.

The equipment captured spectra in the wavelength range of 200 to 900 nm, providing detailed information about the optical properties of the soil samples. The resulting spectra were saved in CSV files, each containing 3501 wavelengths representing the sample's reflectance in the 200 to 900 nm range. These 152 spectra were grouped into a spectral dataset using a script developed in MATLAB R2024a. Fig. 2 shows the spectra corresponding to sample number 1 (100% P_2_O_5_) and sample number 152 (100% soil), while Fig. 3 presents the complete dataset.

### Spectrum preprocessing

The spectral data obtained from the UV–VIS Spectrophotometer were initially exported in Excel CSV format. Subsequently, an application was developed in MATLAB R2022a for the extraction and storage of these data in a .mat file. This stage included the standardization of the spectral values and noise reduction to ensure consistency and accuracy in subsequent analyses.

The data generated by the spectrophotometer showed very high signal fluctuations in the bands between 200 and 250 nm and between 860 and 900 nm, mainly due to noise generated by the measurement limits of the equipment. These noise sources can be found in the light sources, the absorbing medium, the detectors, and the electronic measurement systems used in optical spectrometry [6]. In the rest of the regions, small perturbations are observed across all spectra, indicating the presence of noise, which may be due to the high resolution of the detector that is sufficient to detect very small signal fluctuations [7]. Due to the presence of noise, it is necessary to filter the signals as the first stage of preprocessing. A Savitzky-Golay filter was used for this task. The Savitzky-Golay filter is a data smoothing method used to reduce noise in spectroscopic data and other types of experimental data. The theory behind the filter is based on fitting polynomials to segments of data and then using these polynomials to smooth the data. The Savitzky-Golay filter fits a polynomial of order *n* to a window of size *m* around each data point. The window size must be an odd number, and the polynomial can have an order between 0 and *m* *−* *1*. For each data point, the filter takes a window of neighboring points (of size *m*) and fits a polynomial to these points. The filtered value for the central point is then calculated from the fitted polynomial. The filter has the advantage of smoothing the data while preserving important features, such as peaks and valleys, better than other smoothing methods that might distort these features. The filter coefficients are computed so that the fitted polynomial minimizes the squared error in the data window. These coefficients are used to calculate the smoothed value of each data point [8].

**Fig. 4:**
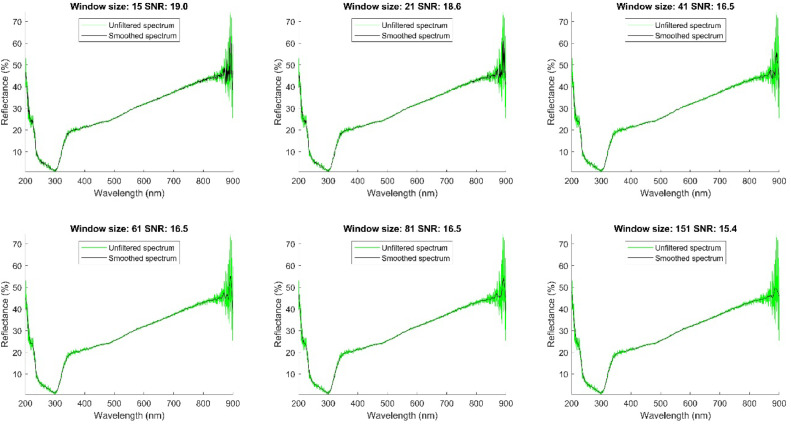
Signal to noise ratio of the filtered spectrum with a polynomial of degree 2 and windows of 15, 21, 41, 61, 81 and 151.

**Fig. 5:**
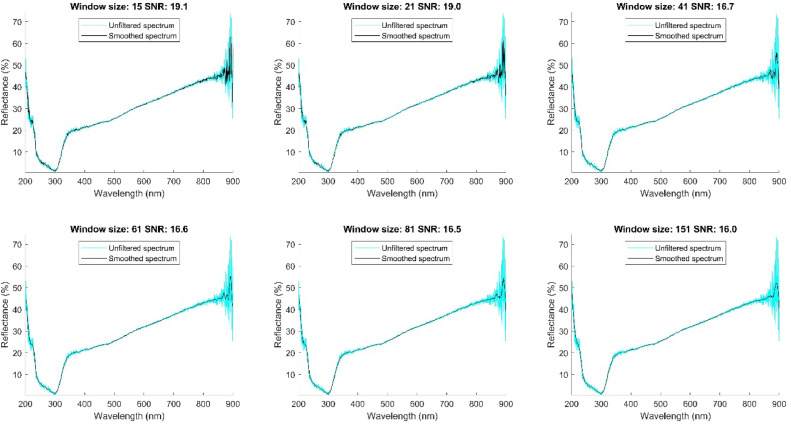
Signal to noise ratio of the filtered spectrum with a polynomial of degree 3 and windows of 15, 21, 41, 61, 81 and 151.

To implement the Savitzky-Golay filter, we considered that the acquisition conditions of the spectra were uniform for all samples, which allowed us to establish a constant noise pattern. On this basis, we adjusted the filter parameters using a single representative spectrum. After applying the filter, we validated its effectiveness by randomly reviewing several filtered spectra, which confirmed that the noise was consistently reduced in all of them. This strategy optimized the filtering process without compromising the quality of the data processing. To tune the filter, we determined the window size and degree of the polynomial as a function of signal-to-noise ratio (SNR) and preservation of spectral information [9]. We initially opted for polynomials of degrees 2 and 3, as they offer a good balance between smoothing and detail preservation. We evaluated window sizes of 15, 21, 41, 61, 81, and 151 points, using the spectrum of sample number 80 to determine the signal-to-noise ratio. Fig. 4 shows the results for a polynomial of degree 2, while Fig. 5 presents the results for a polynomial of degree 3. In both figures, the filtered spectrum is highlighted in black, and the unfiltered spectrum is shown in green and cyan. This procedure allowed us to select the optimal window size, minimizing noise while preserving the integrity of the spectrum, including peaks and valleys that contain relevant information.

We selected the window size based on the signal-to-noise ratio (SNR) close to the average SNR. This criterion ensures that the Savitzky-Golay filter is effective enough to reduce noise while preserving the important features of the spectrum[10]. sing this approach, we chose a window size of 41 points.

**Fig. 6:**
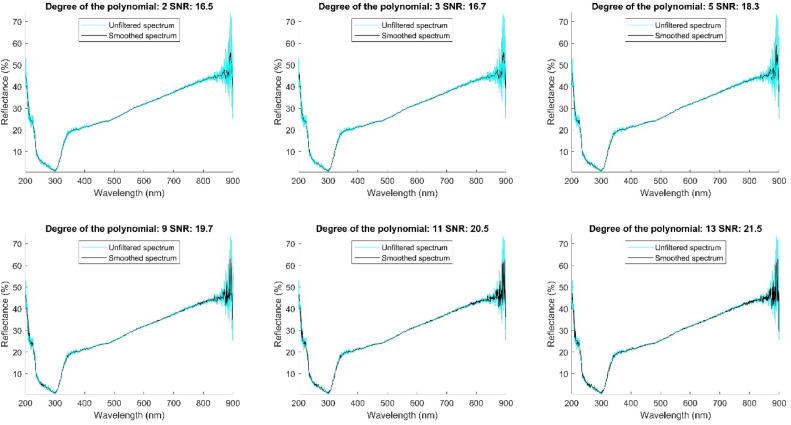
Signal to noise ratio filtered signal with window size 41 and polynomial degree 2, 3,5,9,11,13.

To determine the optimal polynomial degree, we fixed the window size at 41 and evaluated the SNR for polynomials of degrees 3, 5, 9, 11, and 13. Evaluating various polynomial degrees allowed us to identify which provided the best balance between data smoothing and spectral detail preservation. The results of this evaluation are shown in Fig. 6.

In the spectral region near 900 nm, spectra tend to contain a lot of noise, partly because of particle size, especially in soil reflectance measurements, and also because of the measurement limits of the equipment. However, we select polynomials of degrees 2 and 3 in order to smooth the spectrum and retain peaks that may contain useful, if subtle, information. These polynomials attenuate larger fluctuations, probably caused by high-frequency noise, and highlight variations that might be informative. Even so, we recognize that noise remains a challenge in that spectral region.

Among these two polynomials, we selected the degree 3 polynomial due to its higher SNR.

**Fig. 7:**
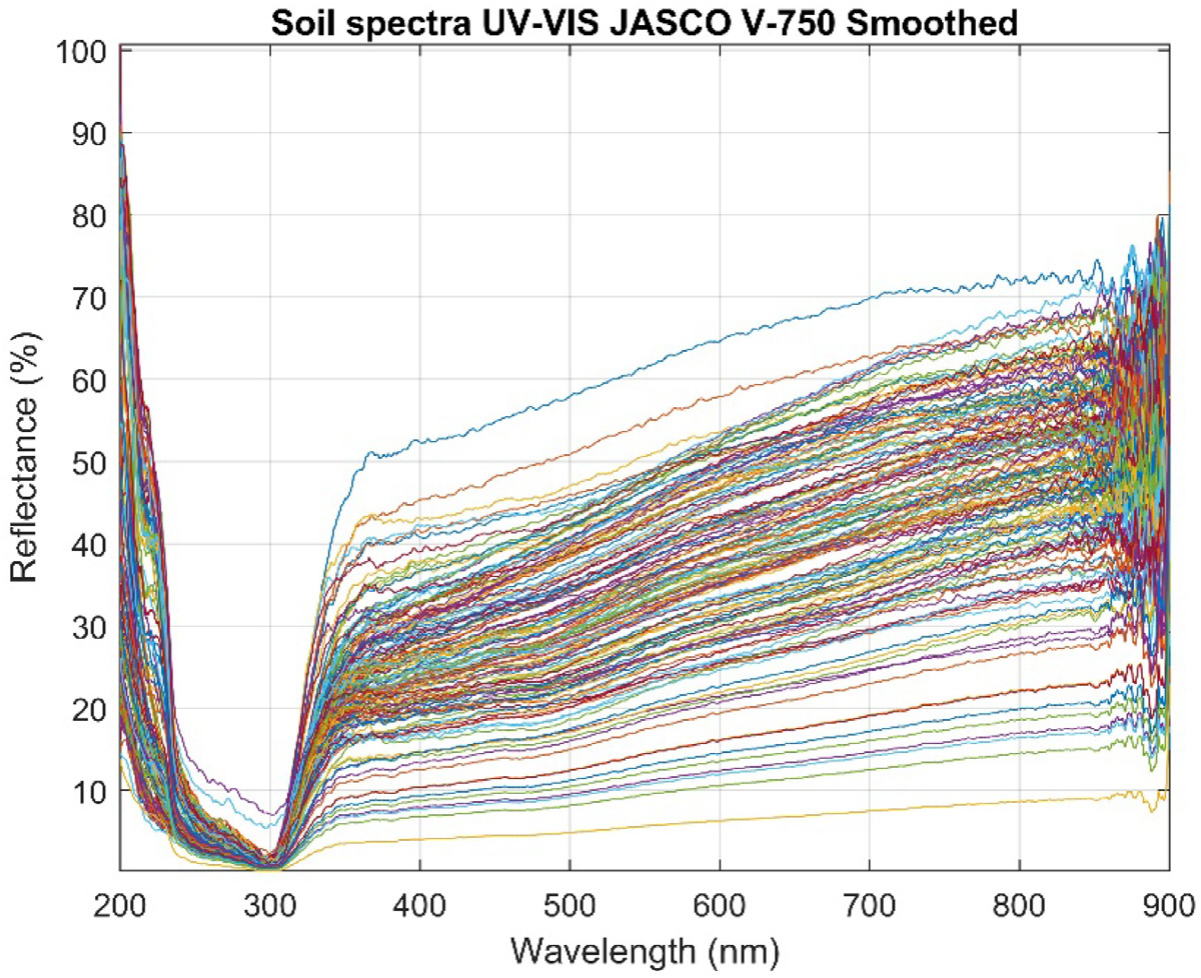
Database filtered with Savitzky-Golay filter.

The 41-point window size with a degree 3 polynomial provides an optimal balance between noise reduction and preservation of significant spectral variations. This balance is crucial for accurately capturing the characteristics of soil and fertilizer. Fig. 7 shows how the filter has improved data quality without losing relevant information.

### Wavelength reduction database

As mentioned above, the spectra obtained from the soil samples contain 3501 wavelengths. To optimize data processing and reduce computational costs, we first identified the most influential wavelengths in the prediction. We used VIP PLS and Random Forest not only as modeling methods, but also because of their inherent ability to assess the importance of features. Although these methods are primarily used to build predictive models, both include mechanisms that allow us to identify and rank the relevance of input variables [[11], [12], [13], [14]]. In this study, we took advantage of these capabilities to reduce the dimensionality of the data set by selecting only the most relevant wavelengths to predict phosphorus concentration. This strategy not only simplified the model, but also improved its interpretability and efficiency. Finally, we combined the bands selected by both methods, removed redundancies and created a new optimized spectral library.

### Variable importance in projection - Partial least squares

VIP-PLS is a technique used for band reduction in spectroscopy. VIP-PLS evaluates the importance of each variable (or spectral band) in the Partial Least Squares (PLS) model, allowing for the selection of the most relevant bands and elimination of the less significant ones [15].

In the first step of reduction using VIP-PLS, we constructed a PLS regression model using all spectral bands. To create the model, we adjusted a key parameter, such as the number of components. Components are latent variables derived from the original variables (predictors and response) that capture as much relevant information as possible. Each component is a linear combination of the original predictor variables. By using only the most important components, PLS reduces the dimensionality of the dataset, improving computational efficiency and the model's generalization capability.

**Fig. 8:**
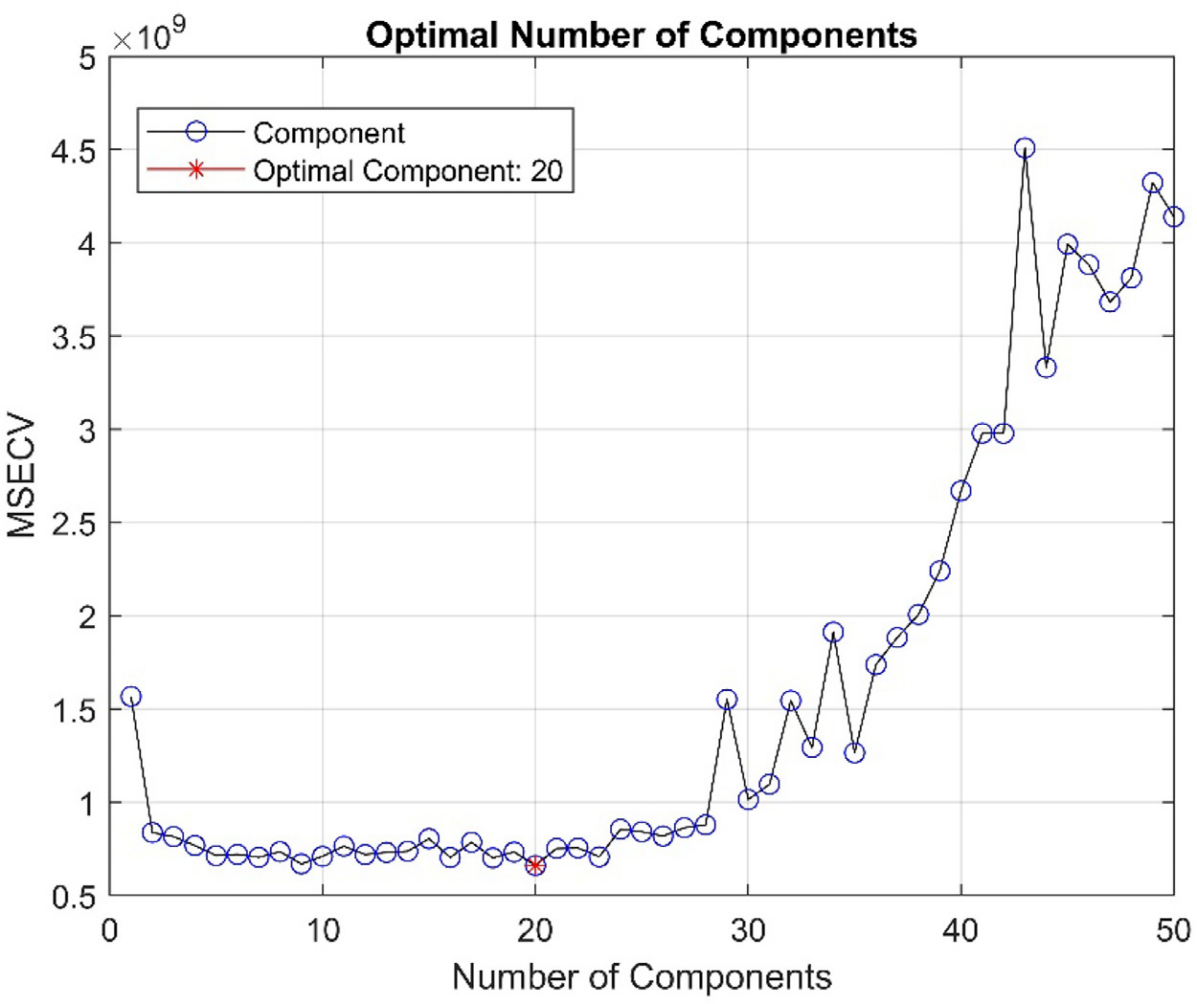
Determination of optimal number of components.

We initially selected 50 components and determined the optimal number of components that minimizes the mean squared error (MSE) [15], as shown in Fig. 8 we observed that the number of components that minimizes the error is 20, and increasing the number of components adversely affects the model.

**Fig. 9:**
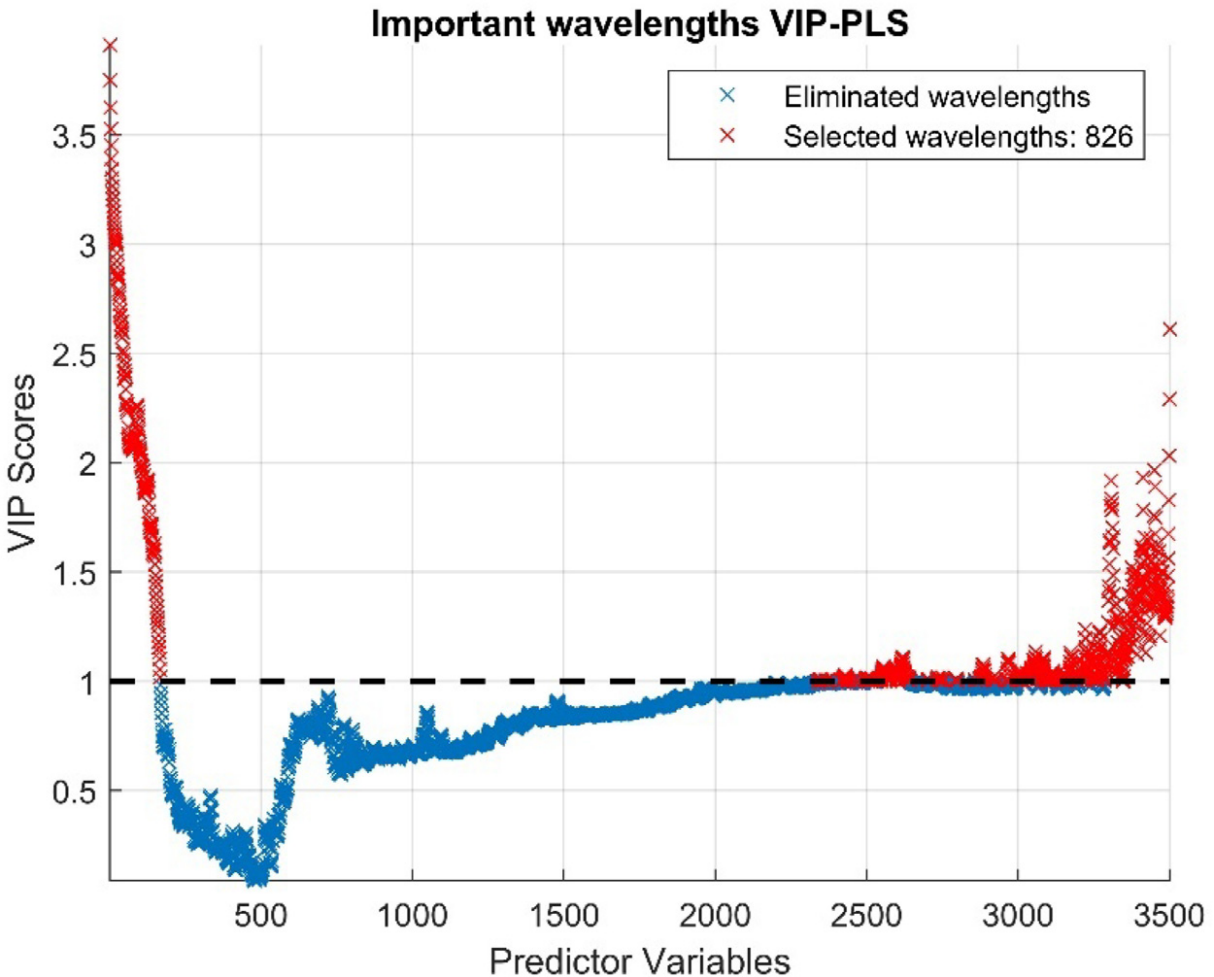
Determining VIP Scores and Significant Wavelengths.

The model adjusted with 20 components allowed us to calculate VIP scores for each band, reflecting their importance in predicting phosphorus content (or any other target variable). We established a threshold of one, which is considered a standard cutoff point for distinguishing between spectral bands that have a significant contribution to the prediction model and those that do not. Using this criterion, we selected the bands with VIP scores higher than the threshold, while eliminating bands with lower scores below the threshold, as they contribute less to the model. In Fig. 9, wavelengths exceeding the threshold are highlighted in red, reducing the number of wavelengths from 3501 to just 826, representing approximately 23.6%.

### Random forest for spectral band reduction

The RF method is a machine learning method used for spectral band reduction in spectroscopy. This method involves building multiple decision trees to evaluate the importance of each spectral band and select the most relevant ones.

First, we constructed a forest of decision trees using bootstrapping, where each tree is trained on a different sample of the data and randomly selects spectral bands at each split. After training, we calculated the importance of each spectral band based on the improvement in node purity. We averaged these importances across all trees to obtain an overall measure of each band's relevance. We selected the bands with the highest importances and eliminated the less relevant ones.

**Fig. 10:**
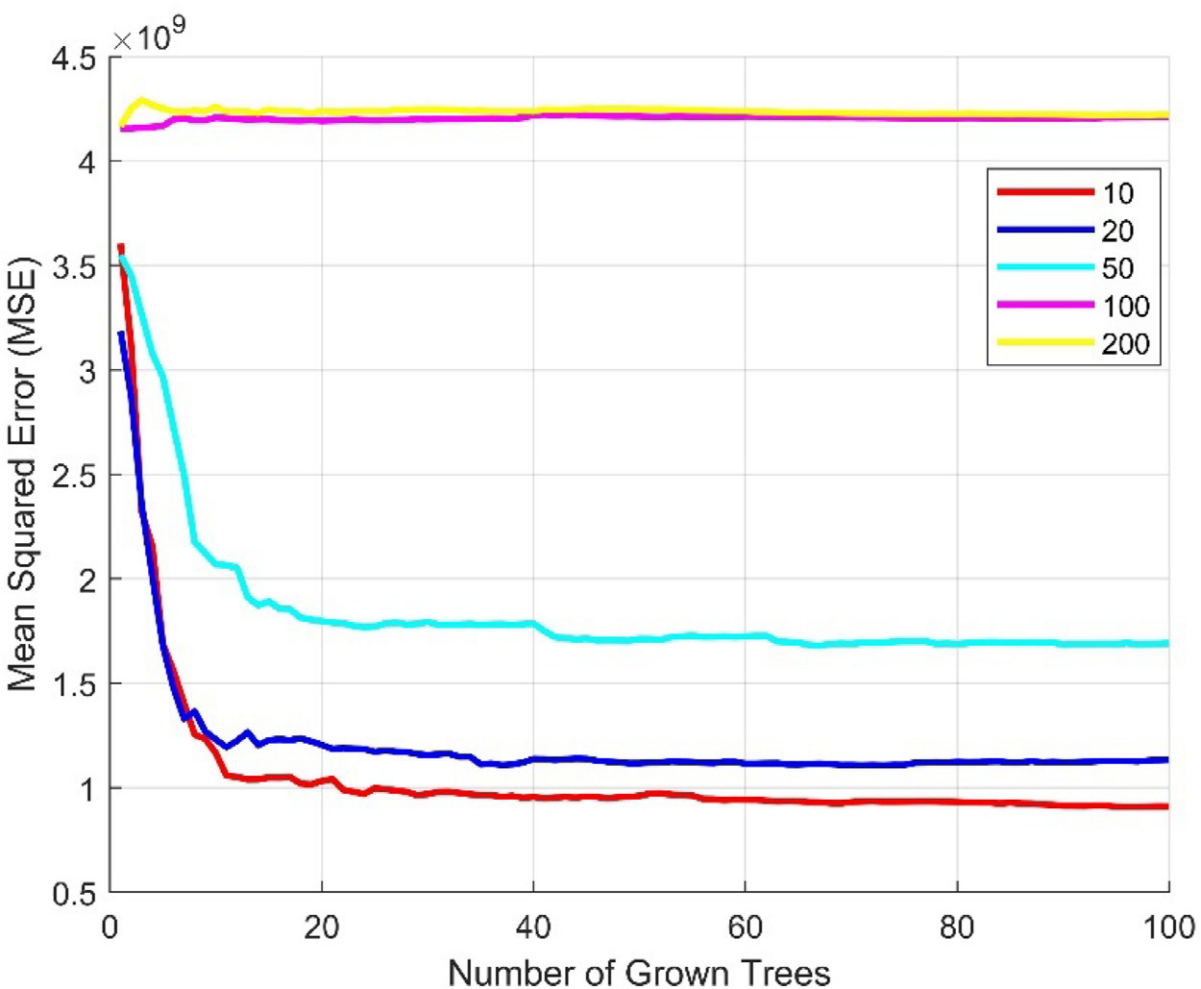
Determining the Optimal Depth of a Random Forest Model with 100 Trees.

To determine the optimal number of trees, we trained the model with different numbers of trees: 100, 150, 200, 250, 300, and 400. We evaluated performance using the mean squared error (MSE) and found that the model provided the best fit with 100 trees. We then fine-tuned the 100-tree model by evaluating the appropriate tree depth, finding that a depth of 10 levels provided the best results, as shown in Fig. 10.

**Fig. 11:**
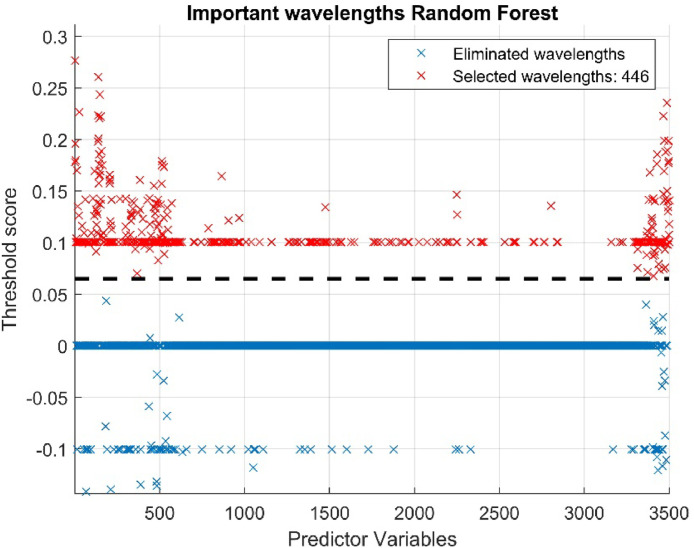
Determining relevant wavelengths in the RF model.

According to Leo Breiman [16], the creator of RF, a sufficiently large number of trees is necessary to ensure a stable estimation of variable importance and good model performance, suggesting that 100 trees or more generally provide a reliable estimate. The results of the band reduction are shown in Fig. 11, where the number of bands has been reduced from 3501 to 446, approximately 13% of the original total data.

### Spectral band reduction and optimized library

The 152 reflectance spectra obtained through spectrophotometry cover 3501 wavelengths. We used VIP-PLS and RF to reduce these bands, resulting in 826 and 446 wavelengths respectively, totaling 1272. From these data, we constructed a spectral library by combining the bands selected by both methods. To ensure that each band in the library was unique, we removed duplicate data, ultimately achieving 1085 wavelengths. The result is a new optimized spectral library that reduces the number of bands from 3501 to only 1085, representing a 69% spectral reduction. This library contains only the most informative and relevant bands, thereby improving the efficiency and accuracy of spectral analysis.

### Dimensionality reduction with PCA

To improve computational efficiency and eliminate redundancies in the spectroscopy data, we implemented dimensionality reduction using Principal Component Analysis (PCA) on the reduced band data. This technique allows us to transform the original data into a new set of uncorrelated variables, known as principal components, which capture most of the variance in the data [17,18]. This method reduces the number of explanatory variables while retaining most of the relevant information. By generating uncorrelated principal components, PCA helps enhance the stability and interpretability of subsequent models. With fewer variables, machine learning algorithms require less time and resources for training.

### Criteria for selecting the number of components

To construct the new reduced-dimensionality dataset, we used the criterion of creating new variables and defined the number of components that explain the variance in the data. In Principal Component Analysis (PCA), we selected components that explain 95% of the total variance of the data. This selection allows us to reduce the dimensionality of the dataset while retaining most of the relevant information. By applying the PCA algorithm to the reduced band data, we obtained the variance explained by each component. In our case study, the results showed that Component 1 explains 70.4% of the variance, Component 2 explains 26.8%, and Component 3 explains only 0.8%, indicating that the first two components account for over 95% of the suggested variance.

According to Hastie [19], this practice is common to ensure that the essential features of the data are captured, eliminating components that mainly represent noise and redundancy. The dimensionality reduction through PCA allowed us to obtain a new dataset with 2 principal components, thus reducing the 152 spectral data that had already been reduced from 3501 bands to 1085 via VIP-PLS and RF to just 2 dimensions. These 2 components contain the most relevant information from the selected wavelengths.

## Method validation

We present a method based on the training and validation of two machine learning algorithms. To achieve optimal performance, these algorithms require high-quality data with relevant information, and their implementation was carried out in MATLAB R2022a.

In the previous section, we explained the process of obtaining, preprocessing, and reducing the dimensionality of the data. Initially, we had 152 spectra, each with 3501 wavelengths. Using Principal Component Analysis (PCA), we reduced these data to 152 samples with only 2 points each. This significant reduction in dimensionality not only preserves the essential information but also considerably decreases computational cost. From there, we implemented two learning algorithms. We trained each algorithm using 80% of the data for training and the remaining 20% for testing. Additionally, we created training sets through 10-fold cross-validation, thus ensuring a more robust and reliable evaluation of the models [20].

### Implementation of feedforward ANN

To make the prediction, we constructed a feedforward artificial neural network (ANN). This network has the capability to model nonlinear relationships and capture complex patterns in the data. With proper regularization and configuration, it can effectively handle large datasets with multiple input variables. Additionally, its high flexibility allows it to adapt to a wide range of prediction problems, making it ideal for use with spectroscopic data. According to Goodfellow [21], feedforward ANNs are widely used due to their ability to approximate complex functions and their effectiveness in modeling high-dimensional data.

The parameter tuning of the ANN was done experimentally by testing different combinations of activation functions and numbers of neurons until the best results were found. During the iterative training procedure, we found that the configuration providing the best results involved two hidden layers. We used the sigmoid activation function in the first hidden layer and the hyperbolic tangent function in the second layer. The sigmoid and hyperbolic tangent functions are common choices that can be effective in different scenarios. Each hidden layer consists of 5 and 4 neurons, respectively.

In the first hidden layer, we chose the sigmoid function because it maps input values to a range of (0, 1). This helps normalize the input data and limits the values to a specific scale, which is particularly useful when the data have high variability. Additionally, the sigmoid function aids the network in learning non-linear features in the input data, which is crucial for capturing complex patterns [22].

In the second hidden layer, we used the hyperbolic tangent function, which maps input values to a range of (−1, 1). Although similar to the sigmoid activation function, the hyperbolic tangent is preferred in deeper layers due to its ability to handle negative values, which helps to center the data. Its symmetry around the origin can also facilitate learning when combined with the sigmoid function from the previous layer.

In the output layer, we employed a linear activation function, which is ideal for regression problems where the task is to predict continuous values. This function allows the output values to take any real value, which is essential for fitting regression data [23].The adjustment of weight and bias values was performed using the Levenberg-Marquardt optimization algorithm. This algorithm updates the weights of the neural network by minimizing the error function [10].

## Results

To evaluate the accuracy of the prediction models, performance metrics such as the Mean Absolute Percentage Error (MAPE), the Root Mean Square Error (RMSE), the Residual Predictive Deviation (RPD) [24], and the Ratio of Performance to Interquartile Range (RPIQ) were used. Tables 4 show the performance evaluation results of the algorithm using these metrics for both the training set and the total dataset.

**Table 4:** ANN feedforward evaluation metrics results.

Feed-forward ANN model		
SET	Test	Total
RPIQ	4.7	4.86
RPD	2.71	2.82
RMSE(g/Kg)	22,991.8	22,845
R²	0.867	0.876
MAPE (%)	19.2	39.4

MAPE measures the size of the percentage error, providing a relative measure of forecasting error that is easy to interpret [25]. It is particularly useful because it expresses errors as a percentage of actual values, which facilitates comparison between different datasets or models. For the training set, values of 19.2% and 39.4% were obtained. Tideswell and colleagues, in 2001 [26], provided an interpretation scale for MAPE, which is used in this research and is recorded in Table 5. According to this scale, a MAPE below 10% indicates excellent accuracy, between 10% and 20% is good, between 20% and 50% is reasonable, and above 50% is poor. This criterion provides a clear benchmark for evaluating prediction quality. Using this interpretation, the model for the training set is classified as having good forecasting performance, while the total dataset is classified as having reasonable forecasting performance.

**Table 5:** MAPE interpretation scales.

MAPE	Interpretation
> 10%	Very accurate predictions
10%−20%	Good forecast
20%−50%	Reasonable forecast
< 50%	Inaccurate forecast

RMSE indicates how far the data points are from a linear regression, that is, it measures the dispersion of residuals or how concentrated the data are around the line of best fit [27]. The RMSE is in the same units as the concentration, which in this case shows errors of approximately 23,000 mg/kg in the prediction for both sets. This is notable because the phosphorus concentration in sample 152, which contains only phosphorus, is just 400.3 mg/kg. When evaluating this error for low concentration values, one might conclude that the predictions have very large errors. However, upon reviewing the trend of the samples, starting with sample 1 which contains 212,571 mg/kg, this value does not seem as large. This suggests that the sample preparation includes very high doping values of P_2_O_5_ compared to the soil sample.

RMSE is especially useful when wanting to understand the absolute magnitude of prediction errors in the same units as the original data. This aspect is crucial for practical applications, as it allows for a direct interpretation of the error in terms of the variable of interest. However, it is important to note that RMSE gives relatively high weight to large errors because the errors are squared before averaging. This means that RMSE is more sensitive to outliers, which can significantly influence the final metric. It is important to consider the specific context of the application; in cases where concentration values are extremely varied, as in this study, it is crucial to understand that an apparently large RMSE may be acceptable if it reflects the true variability of the experimental data.

The RPD evaluates the relationship between the standard deviation (SD) and RMSE. Using this metric to assess the predictive capabilities of the calibration models, values of 2.71 were obtained for the training set and 2.82 for the total set, indicating excellent prediction. According to Nicolaï and colleagues in 2007 [28], predictions evaluated with RPD are classified into ranges. Values between 1.5 and 2 indicate that the model can discriminate between high and low response values. If the indicator ranges between 2 and 2.5, it shows that the model can make approximate quantitative predictions. Values above 2.5 correspond to good to excellent prediction. The obtained values suggest that the model can capture significant variations in the data and providing accurate estimates that can be reliable for agricultural decision-making and soil management.

The RPIQ is a metric that measures algorithm performance by focusing on the quality of predictions relative to the intrinsic variability of the data [29]. It is calculated by dividing the interquartile range by the root mean square error (RMSE). Recently, the relevance of RPD for evaluating prediction accuracy has been debated. According to Bellon-Maurel [30], “RPD uses data variance to penalize the occurrence of outliers in predictions, which tends to decrease as more data become available. On the other hand, RPIQ evaluates the overall quality of predictions in relation to data variability, providing a more comprehensive perspective.”

According to Bellon-Maurel, RPIQ ranges are evaluated similarly to RPD, indicating that values above 2 classify the algorithm as having good prediction accuracy. In the case of this model, RPIQ values of 4.7 and 4.86 indicate that the model has very good prediction accuracy. These high RPIQ values suggest that the algorithm is not only precise but also effectively manages data variability, providing reliable and robust predictions. This metric reinforces confidence in the model and its applicability in practical situations, such as managing and optimizing fertilization in agricultural soils.

**Fig. 12:**
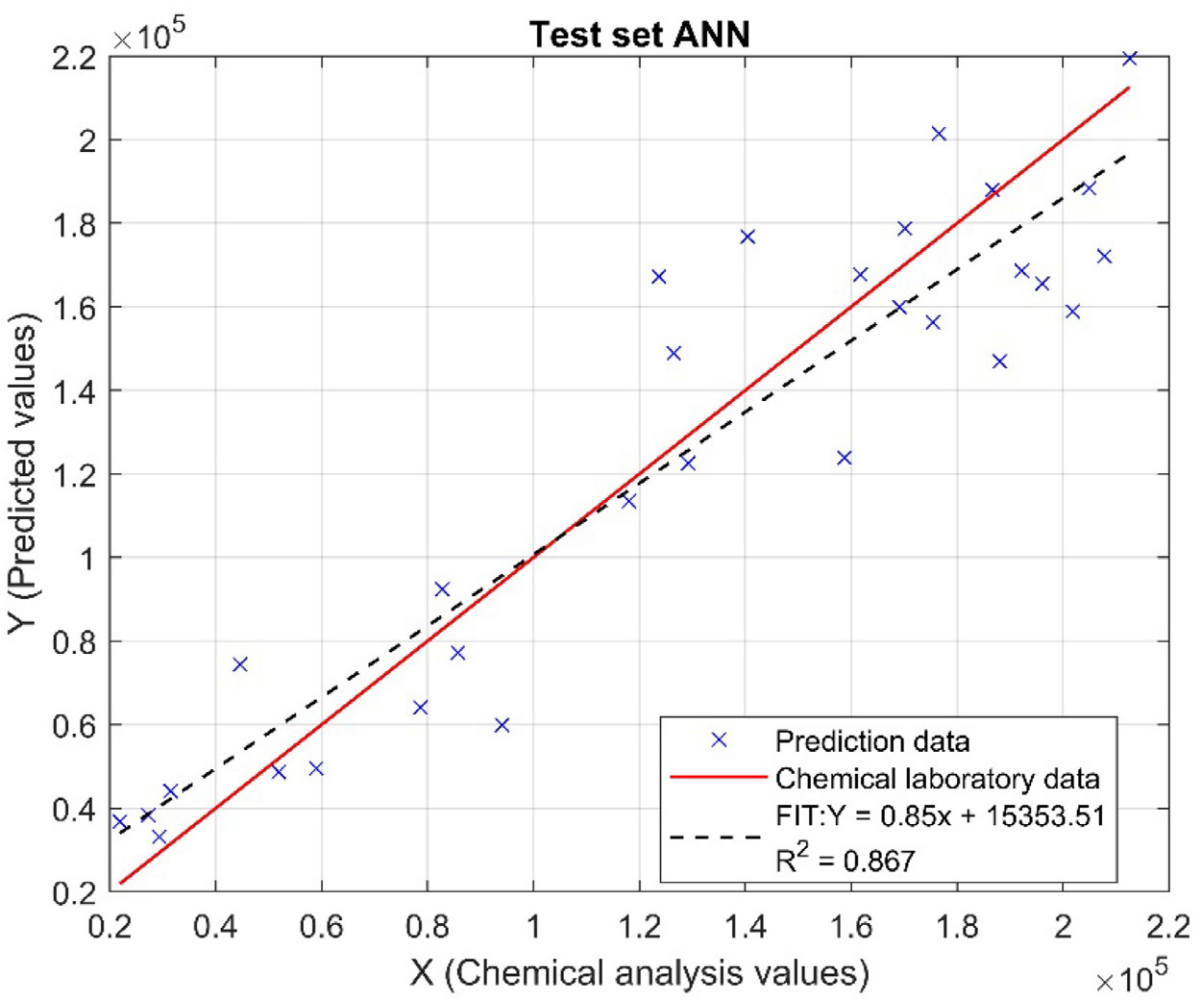
Linear Regression Fit for Test Set: Real vs. Predicted Values (ANN).

**Fig. 13:**
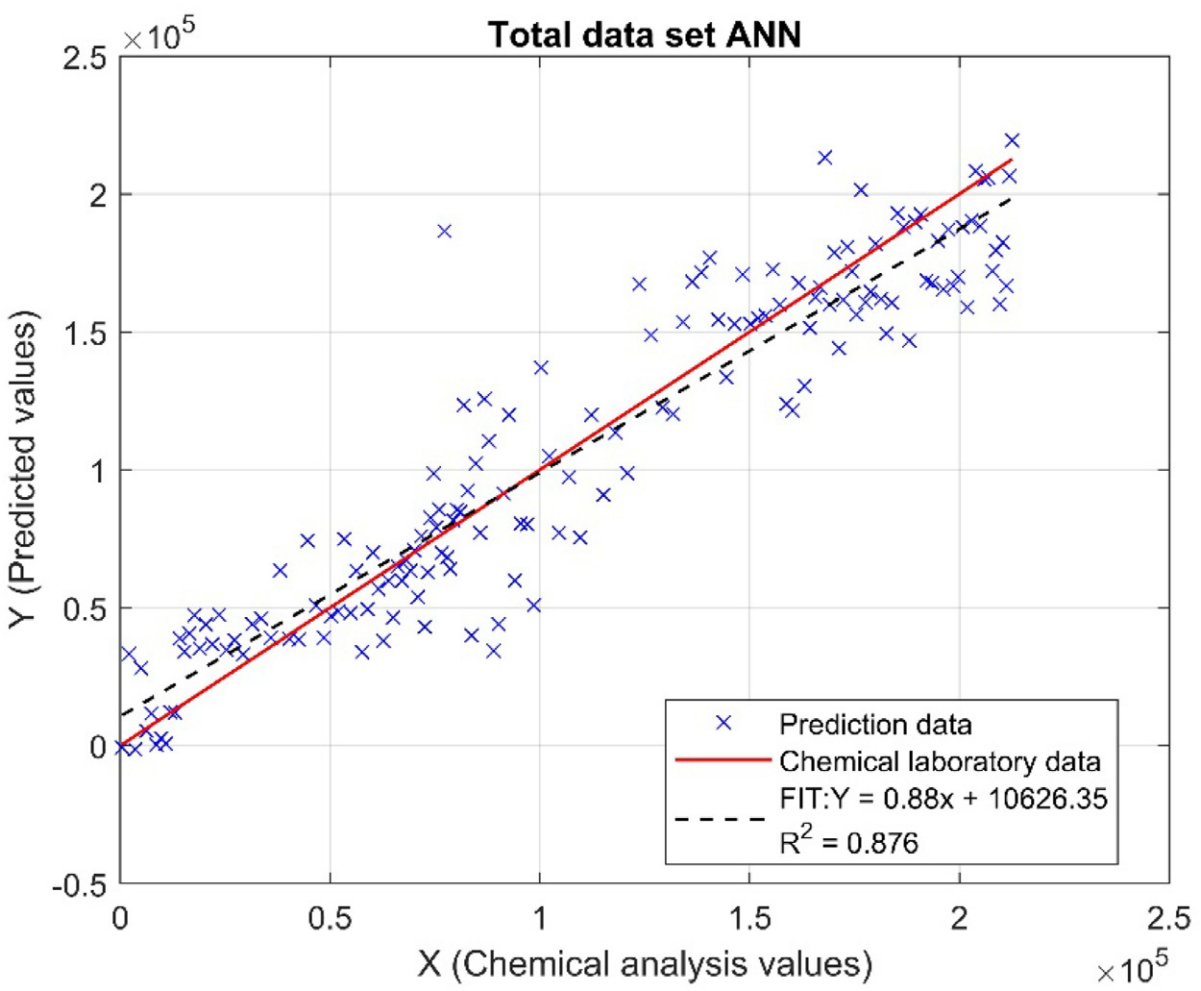
Linear Regression Fit for Total Set: Real vs. Predicted Values (ANN).

The coefficient of determination R^2^ measures the proportion of variability in the response variable that can be explained by the predictor variables in the model. An R^2^ value close to 1 indicates that the model explains the data variability well [31]. The test set achieved an R^2^ of 0.867, meaning that 86.7% of the variability in phosphorus concentration can be explained by the model, indicating a high level of prediction accuracy. Meanwhile, the total set has an R^2^ of 0.876, suggesting that the model has a slightly superior predictive capacity by explaining 87.6% of the total variability. Fig. 12, Fig. 13 show the model's fit for both the test set and the total set.

These results are very promising, as they show that the model using an artificial neural network (ANN) has a good fit for both the test set and the overall set, suggesting that the model is robust and generalizes well to unseen data. Combined with the high RPIQ values (4.7 and 4.86) and an RPD indicating excellent precision, the R² coefficients confirm that the model is effective and reliable for predicting total phosphorus concentration. This suggests that the model can be effectively used in practical applications to improve fertilization management and optimize agricultural production, benefiting both economically and environmentally.

In the study of [32], the ability to estimate phosphorus availability in soil by spectroscopy was evaluated. Two types of soil were used, but the results obtained did not allow effective estimation of phosphorus concentration. The error metrics showed an R² of 0.42 and an RPD of 1.24, indicating very low results. Although the study employed different soil types and data processing methods, the method proposed in our work shows better results despite using only one soil type. Previous studies [33] have used similar techniques, highlighting the use of PLS as a method to predict phosphorus, achieving a coefficient of determination (R²) of 0.72 in the calibration set and 0.87 in the validation set. On the other hand, [34] implements machine learning algorithms and reports comparable results, evaluating with metrics such as R² and RPD, although they do not include RPIQ. In [35], the authors obtain an R² of 0.93 and an RPD of 3.86, slightly higher results, but the study is limited to only 40 soil samples and works in a different spectral range, from 1000 to 2500 nm, while our study covers the range from 200 to 900 nm.

In comparison, the proposed method, which combines VIP-PLS with Random Forest, improves the identification of more relevant wavelengths, which, together with PCA, significantly reduces the computational cost during model training. This optimization decreases the prediction error relative to approaches using PLS alone or linear regressions. Although more algorithms are applied in the process, the resulting model is more efficient in terms of computational resources, making it more suitable for large-scale practical applications.

## Limitations

Uniform sample preparation through grinding and sieving is essential for obtaining accurate results in spectroscopy, as grain size can affect light scattering and absorption, generating noise in measurements. The model was calibrated using a specific type of soil from the Santander region, which limits its applicability to other soil types; to broaden its validity, it is necessary to include samples from various regions and characteristics. Additionally, P_2_O_5_ concentrations in the soil can alter optical properties and affect the accuracy of spectroscopic measurements, so these variations must be considered when developing and calibrating the model.

## Ethics statements

This article did not use animals, humans, or data from social networks.

## CRediT author statement

**Fabio Rivadeneira:** Conceptualization, Methodology, Software, Validation, Formal analysis, Data curation, Investigation, Writing – original draft. **Sandra Nope-Rodriguez:** Conceptualization, Methodology, Supervision, Formal analysis, Software, Writing – review & editing. **Martha Paez:** Methodology, Formal analysis, Supervision, Writing – Original draft, Writing – review & editing, Resources. **Carlos Rafael Pinedo:** Conceptualization, Formal analysis, Writing – review & editing.

## Declaration of competing interest

The authors declare that they have no known competing financial interests or personal relationships that could have appeared to influence the work reported in this paper.

## Data Availability

Data will be made available on request.

## Footnotes

1 https://jascoinc.com/products/spectroscopy/uv-visible-nir-spectrophotometers/models/v-750-uv-visible-spectrophotometer/.

2 https://data.mendeley.com/datasets/fvgswvt5ws/3.
